# Paediatric Meningitis and Hearing Loss in a Developing Country: Exploring the Current Protocols Regarding Audiological Management Following Meningitis

**Published:** 2010

**Authors:** Khoza-Shangase Katijah, Rifkind Romi Emma

**Affiliations:** Box 57, University of the Witwatersrand, WITS 2050

**Keywords:** Paediatric meningitis, hospital audiology referral protocols, audiology referral rates

## Abstract

The purpose of this study was to establish audiology referral protocols for post meningitis paediatric populations in two academic hospitals in Gauteng, South Africa. Specific objectives of this study included determining if audiological assessment referrals were made following infection; determining the time of referral post meningitis diagnosis; establishing what audiological assessments were conducted on this population, as well as determining any correlations between signs and symptoms of meningitis and referrals for audiology assessments. Medical records of 47 children admitted to hospital with a diagnosis of meningitis between the ages of birth and 6 years were reviewed following a retrospective record review design. Data relevant to the current study were obtained from hospital records and this was captured in a data spreadsheet. Both descriptive and inferential statistics were implemented in analysis of the data. Inferential statistics in the form of logistic regression analysis was used to establish any significant factor that may predict referral for audiological assessment. The findings indicated that almost half (40%) of the cases were not referred for audiological services. Of those cases referred for assessment, 89% were referred as in-patients before hospital discharge, with minimal referrals occurring after discharge from hospital. Screening, rather than diagnostic audiology measures were conducted on a majority of the cases. Logistic regression analysis identified fever as the only predictor variable (p<0.01) for audiological assessment referral. Results from this study highlight the need for the establishment of audiology referral protocols for paediatric meningitis populations to ensure that early identification and early intervention occurs.

## Introduction

Internationally, deafness and hearing impairment as a consequence of bacterial meningitis has been reported to be the most common serious complication of bacterial meningitis in the paediatric population; occurring in 6 to 31% of cases depending on the type of meningitis investigated and the type and severity of hearing impairment included in the sample (Brookhouser et al., 1988; Fortnum and Hull, 1992; Dodd et al., 1997; Woolley et al., 1999; Asadi-Pooya et al., 2008). Meningitis is also the most common identifiable cause of acquired profound hearing loss in children and adults (Dodds et al., 1997). Asadi-Pooya, Asadi-Pooya and Rosin (2008) assert that deafness or some degree of hearing impairment occurs in 3.5% to 37% of survivors of meningitis. The incidence may be higher in developing countries than in first world countries since access to resources such as adequate health care is vastly different to that in the developed countries; and also because of the high incidence of HIV/AIDS in these third world countries. Cultural and linguistic diversity influences on health service provision may also be a contributing factor to the incidence and management of meningitis in developing countries, such as South Africa.

It is reported that in developed countries approximately 10% of survivors of bacterial meningitis experience permanent hearing loss while others experience a transient loss of hearing (Richardson et al., 1997). Both permanent and transient losses of hearing are reported to develop in the first few days of illness (Richardson et al., 1997). The onset of the hearing loss associated with bacterial meningitis most likely occurs early in the illness perhaps before other symptoms and signs are evident (Woolley et al., 1999). Furthermore, the phenomenon of fleeting hearing loss may occur in a critical period around the second day of illness during which hearing loss may be reversed (Richardson et al., 1997). This highlights the importance of early referrals and prompt diagnosis which may facilitate performance of appropriate hearing assessment so that hearing loss may be prevented, reversed or further damage is retarded (Woolley et al., 1999).

Research by Woolley et al. (1999) indicates that, in majority of cases, if hearing loss is present at discharge from hospital, recovery of hearing does not occur. Therefore, referral of patients for audiological services is essential in order for effective early intervention services to be provided. “All children recovering from bacterial meningitis should be referred for audiologic assessment before discharge from the hospital” [4 p. 513]. This referral is recommended by some authors to be appropriate only 2 to 4 weeks after discharge to allow for any middle ear involvement to resolve (Dodds et al., 1997), and to ensure that profound hearing impairments are detected early enough to enable a cochlear implant to be a viable option (Fortnum and Hull, 1992). However, hearing loss post meningitis has been found to fluctuate or deteriorate over time and therefore it may be insufficient to assess hearing only once after discharge (Koomen et al., 2003). Nevertheless, the importance of early identification and monitoring of this hearing impairment is highlighted.

In a retrospective audit performed by Riordan et al. (1993), the major reason for hearing not being assessed in this population was lack of referral. This is different to findings by Fortnum and Hull (1992) which indicated that, in their study, reasons for non-referral included factors such as the belief held by some paediatricians that it is generally unnecessary to do formal hearing tests following bacterial meningitis; the assertion that only in children where there was already some concern over their hearing should be referred; and the belief that only children who met some other criterion should have their hearing tested. Riordan et al. (1993) suggested that routine referral for audiological assessment at discharge in conjunction with re-referral at out-patient attendance may assist in increasing the number of children assessed after bacterial meningitis, while Fortnum and Hull (1992) asserted that to refer postmeningitic children for hearing testing should not be in question, but that referral should be made while the child is still in hospital and that the importance of the assessment be emphasized to the parents. In a qualitative analysis of parents' experiences with early detection of hearing loss, research found that powerful emotions experienced by the parents at diagnosis included denial and shock as well as frustration arising from the delays in diagnosis (Russ et al., 2004), hence early referrals are of paramount importance to the emotional adjustment as well as compliance to treatment strategies of the parents. More recently, Koomen et al. (2003) advocate that hearing loss following meningitis can be predicted satisfactorily, hence when the hearing of children who are predicted to be at risk is tested as part of their routine follow-up, no children with hearing loss need be missed.

The nature of the hearing loss in postmeningitic children is most commonly sensorineural (Richardson et al., 1997), with the exact mechanism of the hearing loss still not very well understood. However, the mechanism is thought to be likely due to multiple factors which include direct labyrinth involvement, cochlear neuroepithelial damage, and vascular insult (Kutz et al., 2006). There is some evidence indicating possible lesions to the brainstem or higher cortical areas, but the site of auditory lesion as a result of damage following meningitis is almost always to the cochlear and the organ of corti, thus providing exclusion of damage to the cochlear nerve (Richardson et al., 1997). This survival of the cochlear nerve makes these children good candidates for cochlear implantation (Richardson et al., 1997). However, Wilson et al., 2003) describe how the cochlear duct may be obliterated by osteoneogenesis within a few months of meningitis which may make cochlear implantation ineffective or impossible. Therefore, early identification is vital (Dodds et al., 1997).

Failure to detect hearing loss may result in significant consequences for the child's speech and language acquisition, academic performance as well as social and emotional wellbeing. Therefore the identification of hearing loss in these children is crucial in order to ensure early habilitation and minimising of the long term educational, social and emotional difficulties that these children may experience (Bush, 2003; Kutz et al., 2006). Research has shown that children who are deprived of sufficient amounts and/or quality of language input in their earliest years are at risk for poor outcomes in both language and academic endeavours later in childhood (Nicholas and Geers, 2006). In addition, children detected late may never catch up with their normal hearing peers in academic, social and emotional domains of development (Olusanya, 2008). On the other hand, evidence in developed countries indicate that early hearing detection and intervention programs leads to linguistic, speech and cognitive development that is comparable to normal hearing peers (Hatzopoulos et al., 2007). Thus, effectively addressing the developmental constraints commonly associated with childhood hearing loss becomes crucial.

When investigating the impact of hearing loss following meningitis, deficits in the domain of speech and language development become clearly evident. Research by Nicholas and Geer (2006) on the effects of early auditory experience on the spoken language of deaf children illustrated that profoundly deaf children demonstrated a much lower level of lexical and syntactic skills. This low level of lexical and grammatical proficiency places profoundly deaf children at a disadvantage when compared to their chronologic age-mates. This results in the inability of these children to fully participate in, and benefit from typical preschool activities without a high degree of communicative support (Nicholas and Geers, 2006). These speech-language difficulties may be further exacerbated beyond childhood, as these individuals may not be accepted, with a lack of effort being made to communicate with them. They may also demonstrate poor involvement in social activities due to a fear of stigmatisation or rejection by community (Hatzopoulos et al., 2007; Moeller, 2000; Olusanya, 2008; Yoshinaga-Itano, 2004).

A follow up study performed by Bedford et al. (2001) at five years post meningitis indicated severe communication problems or absence of speech in 55 of 1584 subjects, with a further 9.4 % of the children having speech or language delay. Research by Woolley et al. (1999) indicated that 21% of school age survivors of meningitis had functionally important disabilities including central auditory perceptual dysfunction that adversely affects learning ability. Decreased academic performance and behavioural disabilities were also indicated by these authors. Had intervention been implemented early following the diagnosis of meningitis, these long-term sequelae could have been prevented or minimised.

In an Australian study tracking 12 year outcomes following bacterial meningitis, results indicated that these children remain at significant risk for neurological abnormalities (Grimwood et al., 2000). Central auditory function was found to have improved with time, however, difficulty with language based tasks remained. In the same study, findings regarding audiological outcomes indicated that while most sensorineural hearing loss following meningitis remains stable, spontaneous fluctuations or even progression may occur more than twelve years after recovery (Grimwood et al., 2000). These findings raise the need for audiologists to be involved in the initial diagnosis and management, as well as long term monitoring of post meningitis paediatric populations. By ensuring that early diagnosis and early intervention occurs these difficulties may be averted or at least appropriate intervention may be provided thus decreasing the negative impact of disability in this population group. This early diagnosis can only be achieved if sensitive and comprehensive audiological tests form part of the assessment and management protocol of these patients.

Research indicates that otoacoustic emissions (OAEs) combined with tympanometry followed by an auditory brainstem response (ABR) if indicated is the best test battery that should form part of hearing evaluation guidelines and protocols for this population (Woolley et al., 1999). Behavioural audiometry is also recommended as soon as possible in order to establish the degree and configuration of the hearing loss (Woolley et al., 1999). In a study by Faraji et al. (2004) ABR, OAE and Behavioural Audiometry performed in children with meningitis during the critical period and recovery period was found to diagnose any degree of hearing loss with a high level of accuracy. The efficacy of OAEs as a screening tool for children recovering from acute bacterial meningitis was researched by Richardson, Williamson, Reid, Tarlow and Rudd (1998), and findings indicated that OAE screening was both feasible and effective. OAEs were found to be highly sensitive and relatively specific. Furthermore the technique was found to be well tolerated by patients. Therefore, in-patient OAE screening has been identified as a method of early diagnosis of hearing loss and prompt auditory rehabilitation (Richardson et al., 1998). However, it must be considered that retro-cochlear hearing loss may be missed by an OAE screening as the pathologic lesion is in the auditory nerve or higher centre (Richardson et al., 1998). This highlights the importance of cross check principles and holistic test batteries used in audiology diagnosis.

Audiologists should also be aware of the symptomatology that occurs during meningitis as these may be useful to take into consideration when examining risk factors for paediatric populations who may form part of their case load. Information regarding symptomatology experienced during meningitis is of great importance as Kutz et al (2006) identified factors such as the length of hospitalization, development of seizures, fever, elevated cerebrospinal fluid protein and decreased cerebrospinal fluid glucose as significant predictors for hearing loss in children with bacterial meningitis. Grimwood et al. (1996) identified that age (children under 12 months), seizures developing or persisting after 72 hrs of appropriate treatment in hospital, and the presence of focal neurological signs are each important risk factors for adverse outcomes of bacterial meningitis.

By identifying and acknowledging symptomatology during illness professionals may identify factors associated with long term sequelae. In doing so the professionals may ensure appropriate additional follow up treatment as well as parental counselling Grimwood et al., 1996). Therefore, having knowledge of these prediction factors may be useful when establishing referral protocols for audiology assessments. In order to identify children with meningitis who have acquired a hearing loss it is recommended that all children at risk have a reliable evaluation of their hearing. Referral for a hearing assessment should be an essential part of the treatment protocol for all such children. As postulated by Wilson, Roberts and Stephens (2003), a well defined referral pathway can improve assessment and referral rates post meningitis.

Despite South Africa having a relatively well-developed infrastructure compared to other regions in Sub-Saharan Africa, wide scale access to health care (including audiological services) for the majority of its citizens is far from common practice. Universal newborn hearing screening and routine hearing testing of children who present with risk factors for hearing loss is not performed as part of routine paediatric management. This may be due to the limited state resources, but may also be due to the lack of contextual research on paediatric hearing impairments. The high incidence of HIV/AIDS, which presents as an overwhelming burden to the health resources of the country, may also be a factor in that available resources may be directed toward management of HIV/AIDS at the possible expense of other conditions. The fact that meningitis is one of the main opportunistic infections seen with HIV/AIDS, its general management requires investigating; and that includes management of the known consequences of meningitis, including hearing impairment. Therefore research in a South African context is vital for the collation and development of appropriate and efficient hearing evaluation guidelines and protocols for the post meningitis population, which highlights once more the importance of this study.

## Method

### Aim of the study

The primary aim of this study was to establish what the audiological referral protocols are for post meningitis paediatric populations in Johannesburg, Gauteng. There were four specific sub-aims to the study:

1. To determine if audiological assessment referrals were made following infection.
2. To determine the time of referral post meningitis diagnosis.
3. To determine what audiological assessments were conducted on this population.
4. To determine if a correlation exists between the presenting signs and symptoms and the audiology referrals

### Design of the Study

This study was a record review and was therefore retrospective in nature. The adopted design allowed for examination of data already on file, which meant that the researchers could make observations and provide descriptive statistics from this data (Schiavetti and Metz, 2006). Record reviews are useful in instances where it is logistically infeasible to conduct an experiment relating the variables of interest (McBurney & White, 2007), as in the current study. However, an acknowledged limitation of this design is that the researcher is limited to the information exclusively provided by the agency that collected the data and by any biases or inaccuracies present in the collection procedure (McBurney & White, 2007).

### Participants

#### Participant selection criteria - Inclusion Criteria

The participant inclusion criteria included the following:

- All files reviewed had to be of children between the ages of birth to 6 years as the focus was on early intervention.
- All files reviewed had to be of children who were admitted to the hospital and treated at the hospital for meningitis. Files included records which dated back to 3 years to ensure that the findings would not be influenced by short term factors such as staffing shortages, strikes, and so on.
- All files reviewed had to be of children who had normal hearing function pre-morbidly, based on the case history data in the files.

#### Participant selection criteria - Exclusion Criteria

The participant exclusion criteria included the following:

- Admission for conditions other than meningitis.
- Pre-morbid hearing impairment.
- Age older than 6 years.

#### Sampling Procedure

A non probability convenience sampling technique was adopted in the current study since the sample was restricted to a part of the population that was readily available and random sampling would have been difficult to achieve (Schiavetti and Metz, 2006). Participants were recruited by consulting the paediatric medical ward discharge summaries. These discharge summaries provided hospital codes which allowed for full medical records including bed letters to be obtained. Consequently, clinical information for review was obtained both from discharge summaries as well as from full medical records. Participants with the diagnosis of meningitis were recruited to participate in the study.

#### Participant Description

A total of 100 participants with a positive history of meningitis including both males and females comprised the research sample at the initial stages of this study. Of these 100 participants only 47 passed the inclusion criteria stipulated above and therefore comprised the final sample size. These participants were from two tertiary academic state hospitals that had both paediatric medical wards as well as Audiology departments.

#### Research procedures and materials

All files that met the inclusion criteria were reviewed and data was captured on a spreadsheet. The spreadsheet was designed by the researchers with the aim of capturing the following data:

- **Hospital Code** (A or B)
- **Patient code number**
- **Age:** The age of the child at the time of infection is of vital importance as evidence suggests that there is a critical, or at least a sensitive, period for language acquisition (Hurford, 1991).
- **Gender**
- **Type of meningitis:** In a study by Kutz et al. (2006) on the clinical predictors for hearing loss in children with bacterial meningitis, the incidence of hearing loss was greater in patients with Streptococcus Pneumoniae meningitis than in patients with Neisseria Meningitidis thus illustrating the importance of establishing the type of meningitis.
- **Symptomatology:** By identifying and characterising symptomatology during illness, professionals may identify factors associated with long term sequelae following meningitis. In doing so the professionals may ensure appropriate additional follow up treatment as well as parental counselling (Grimwood et al., 1996). Therefore, symptomatology was included in the data spreadsheet.
- **Treatment**
- **Duration of hospital stay:** Research by Kutz et al. (2006) identified the length of hospitalization as a significant predictor for hearing loss in children with bacterial meningitis.
- **Was audiological assessment recommended?**
- **Time of referral**
- **Type of audiological tests conducted**
- **Other referrals made:** The collaboration among professionals and families in the effective delivery of service to infants and children with hearing loss is imperative in ensuring appropriate services are received. Therefore, other referrals were included in the data spreadsheet in order to ensure this information was captured.

#### Validity and reliability

The information collated for the current study was thought to be fairly reliable since this information was obtained from medical records as opposed to patient self reports. Turkkan (1999) asserts that even straightforward questioning on self reports is fraught with potential for misunderstandings thus illustrating a limitation of self reports. Furthermore, special ethical considerations need to be taken into account during self report data collection as individuals may not be fully capable of understanding the research protocols (Turkkan, 1999). The researcher reviewed the files personally to ensure that errors in capturing were eliminated, and half of the files were re-reviewed by the co-researcher to ascertain reliability of the data captured. External validity of the current study was however compromised by the fact that systematic randomised sampling was not the procedure used, and the data was only pooled from two hospitals in Gauteng.

#### Data analysis and statistical procedures

This study made use of descriptive and inferential statistics. Descriptive statistics was used as it allows for group differences to be observed; and developmental trends or relationships among variables to be measured by the researcher (Schiavetti and Metz, 2006). Inferential statistics was also adopted as it makes use of mathematical methods (such as hypothesis development) that employ probability theory for deducing/ inferring the properties of a population from the analysis of the properties of a set of data drawn from it (Fife-Shaw, 2002). Inferential statistics, in the form of regression analysis, was conducted (McBurney & White, 2007). Logistic regression was chosen as audiological assessment in the current study is a binary response variable (i.e. the child was either referred or not) and the predictor variables were a mix of discrete and continuous variables (Fife-Shaw, 2002). Age, gender, fever, headache, stiff neck, vomiting, stomach ache, dizziness, cough, seizure, duration of hospital stay, and type of meningitis were the variables assessed. The null hypothesis for the current study was that there is no relationship between these variables and referral for audiology services, while the alternative hypothesis was that at least one of these factors is related to whether or not an audiology referral is made. To statistically test the hypothesis a significance level (alpha) of 0.05 was selected which meant that there would be a 95% confidence level that results were not due to chance (Howell, 2007).

#### Ethical consideration

Ethical clearance was obtained from the University of the Witwatersrand, Human Research Ethics Committee (medical) before the study was conducted (protocol number: M080345). The researchers ensured that permission was obtained from the relevant authorities (hospital superintendents and heads of the wards) at the research sites. Written informed consent to participate in the study was obtained before the record reviews were conducted with an assurance that confidentiality of all records would be maintained. Furthermore, to ensure anonymity, researchers ensured that no personal or identifying information was included in the research report and research coding numbers instead of identifying information were used. The current study also reduced risks to the participants to a minimum since it was a record review. Lastly, the hospitals involved were given the opportunity to request to see the research results if they were interested (South African Medical Research Council, 2008).

## Results

The results obtained from this study are presented in accordance with the specific aims of the study. It should be noted that the results are not discussed separately for the two participating hospitals because the researchers believed that no additional information would be gained from that process, particularly because of the final sample size. A demographic profile of the participants is displayed in Table 1.

**Table 1:** Demographic profile of all participants in the study (N=47)

Factor	Sub-Category	Number	Percentage
Age Range	4days to 6 years	47	(mean age: 1.9years)
Gender	Male Female	25 22	53% 47%

There were 47 participants in the study (as seen in Table 1) ranging in age from 4 days to 6 years with a mean age of 1.9 years. Participants included both males and females.

As illustrated in Table 2, various forms of meningitis were identified in the current study with meningococcal meningitis being the most prevalent with 45% of the sample presenting with this form, while Haemophilus. H influenzae was the least presenting type of meningitis (4%).

**Table 2:** Medical profile of all participants in the study (N=47)

Factor	Sub-Category	Number	Percentage
**Type of meningitis**	Meningococcal Strep. Pneumonia Group B Strep Tuberculosis Haemophilus H. Influenza Bacterial Unspecified Pathogen	21 5 5 5 2 2 7	45% 11% 11% 11% 4% 4% 15%
**Presenting** **symptomatology**	Fever Vomiting Seizures Stiff neck Diarrhoea Stomach cramps Bulging fontanel Cough Irritability Headache Increased tone Inability to sit/walk Oral candida Oral ulcers Photophobia	28 19 18 17 11 10 9 7 6 5 4 2 1 1 1	**N/A** **N/A** **N/A** **N/A** **N/A** **N/A** **N/A** **N/A** **N/A** **N/A** **N/A** **N/A** **N/A** **N/A** **N/A**
**Duration of** **Hospital stay**	Range Average Standard deviation	5–21 days 10.3 days 4.4 days	**N/A**

Key: N/A=not applicable

The most commonly presenting symptomatology in the current sample (Table 2) included fever, vomiting, seizures and neck stiffness, with the duration of hospital stay ranging from 5 days to 21 days (with an average of 10.3 days).

### Audiology referrals made

The first objective of this study was to determine if audiological assessment referrals were made following meningitis. Findings from the current sample indicated that referrals were made in 28 cases indicating a referral rate of 60% as depicted in Table 3. Almost half (40%) of the cases reviewed were not referred.

**Table 3:** Audiological referral profile of all participants in the study (N=47)

Factor	Sub-Category	Number	Percentage
**Was the referral** **made?**	Yes No	28 19	60% 40%
**Time of referral** **(n=28)**	As in-patients As out-patients soon after discharge Later than 3 months	25 3 0	89% 11% 0%
**What audiological** **services were** **conducted?** **(n=28)**	Otoscopy Tympanometry Basic Pure tone audiometry Speech Audiometry OAEs ABR ASSR	28 28 0 0 9 0 0	**N/A** **N/A** **N/A** **N/A** **N/A** **N/A** **N/A**
**Other referrals**	Physiotherapy Occupational therapy Speech-Language Therapy Dietician Follow-up clinic	8 4 4 3 4	**N/A** **N/A** **N/A** **N/A** **N/A**

Key: N/A=not applicable

### Time of referral for audiology testing

The second objective of this study was to determine the time of audiological referral in relation to the onset of infection. Of the 28 cases that were referred, 25 (89%) were referred as in-patients before discharge from the hospital, with the other 3 cases being referred after discharge (but within three months). It must be noted that in the sample reviewed, no cases were referred three months post discharge and onwards (Table 3).

### Audiological assessments conducted

The third aim of this study was to establish which audiological tests were conducted on this population. Of the 28 cases referred for audiological assessment (Table 3), all cases had otoscopic examinations and tympanometry conducted, and only 9 cases had OAEs performed. No participants had basic pure tone audiometry, speech audiometry, auditory brainstem response, and auditory steady state response.

### Correlation between signs and symptoms and audiology referrals

The final aim of the study was to determine if there was any correlation between signs and symptoms and audiology referrals. Logistic regression was utilized to determine what predictive factors can be used to model whether or not a child will be sent for an audiological assessment based on symptomatology. Of all the variables analysed, the only significant predictor variable where p< 0.01 was fever (whether the child presented with it or not). This infers that if one were to model predictors that could determine whether a child will be referred for an audiological assessment, fever was the only variable indicator. This finding can be supported within the current study, particularly since it is consistent with previous findings on fever in meningitis being a predictive factor for hearing loss. However, because of the small sample size in the current study, this finding cannot be put forward as the only recommendation to be followed in post meningitis hearing referral. Although fever can be considered as a variable indicator, other indicators should still be used to ensure that no cases of post meningitic hearing loss are missed. Nevertheless, current findings indicate that fever must be acknowledged as a crucial factor to be included during the development of audiology referral protocols post meningitis.

### Additional Analysis

#### Length of Hospital Stay

The average duration of hospital stay in the current study was 10.3 days ±4.4 days. The shortest duration of stay was 5 days and the longest duration of stay was 21 days.

#### Other Referrals

Although the audiologist is typically the first professional to identify hearing loss in infants and children, other professionals may also identify risk factors for hearing loss. Therefore, information regarding referrals to other professionals was included in this study. Eight children were referred for physical therapy, four children were referred for occupational therapy, four children were referred for speech-language therapy, four children were referred to a follow up clinic and three children were referred to a dietician as depicted in Table 3.

## Discussion

As can be seen in Table 1, the sample for the current study seemed to be fairly representative of the South African paediatric population infected with meningitis as there were slightly more males than females in the sample; as seen in the reported distribution of meningococcal meningitis by gender in South Africa between the year 2001 and 2005 (Department of Health: Republic of South Africa, 2006). Furthermore, the fact that this sample consisted of 34 cases (72%) under the age of three years is consistent with data released by the South African Department of Health (Department of Health: Republic of South Africa, 2006) which indicate that in South Africa between 2001 and 2004, 46% of notified cases of meningitis with known age were children under the age of five years. This age of presentation highlights the need for more vigilant audiologic management of these cases as this is the critical period for speech and language development. Furthermore, this is the stage when cognitive skills are still immature, and hence undetected deficits post meningitis may remain until the child has started school; which is not of benefit to that child (Grimwood et al., 1996).

Meningococcal meningitis was the most commonly presenting meningitis with a positive diagnosis in 21 children (45%), with Haemophilus. H influenzae being the least presenting type of meningitis (4%) (Refer to Table 1). These findings are consistent with those found in Athens, Greece; where meningococcal meningitis was also the leading type of meningitis with 66% of the sample presenting with it (Theodoridou et al., 2007). Findings from the current study seem to imply that pathogens causing meningitis in South Africa may be similar to pathogens causing meningitis in other countries; possibly indicating the probability that the burden of disease might be experienced in a similar fashion; although debatably co-existence of other infectious diseases may be much higher in this context. This has great implications for the audiological management of this population as this indicates that adoption of management protocols established in developed countries may be appropriate for the management of the South African paediatric meningitis population.

The most commonly occurring presenting symptoms in the current study were fever, vomiting, seizures and stiff neck. These are the classical features which are often documented in meningitis (Van de Beek et al., 2004). Again findings of the current study are consistent with the findings of a study by Valmari et al. (1987) in Finland and confirm that the sample for the current study seems to be representative of the general meningitis population, although Wubbel and McCracken (1998) suggest that presenting symptomatology of meningitis may differ according to the age of the individual as well as the type of meningitis. Information regarding symptomatology experienced during meningitis is of great importance as it was identified that age (that is children under 12 months), seizures developing or persisting after 72 hrs of appropriate treatment in hospital and the presence of focal neurological signs are each important risk factors for adverse outcomes of bacterial meningitis (Grimwood et al., 1996). Findings from the current study on symptomatology are particularly important since these symptoms have been identified as significant predictors for hearing loss in children with bacterial meningitis (Kutz et al., 2006). Knowledge of these predictive factors by the health care team involved in management of paediatric meningitis will not only facilitate the establishment of audiological referral protocols for this population; but may also aid professionals in identifying factors associated with long term sequelae. In doing so, these professionals may ensure that appropriate and comprehensive follow up treatment as well as parental counselling is in place for families that need it (Grimwood et al., 1996).

As far as referral for audiology services is concerned, the current study revealed that almost half (40%) of the cases were not referred for audiological assessment following meningitis. This is significantly higher than reports from developed countries such as England; where although some cases are not referred, only less than a quarter of these cases were not referred. Wilson, Roberts and Stephens (2003) indicated that between 7.7% and 27% of children in Wales were not referred for hearing testing post meningitis. In a study by Riordan et al. (1993) in England 75% of survivors of bacterial meningitis received audiological assessment between 1998 and 1999, a number that is significantly higher than that reflected in the current study. Riordan et al. (1993) identified that in their context, the major reason for children not being assessed was non-referral. Although reasons for children not being assessed were not explored in the current study, it is possible that non-referral also played a significant role. Over and above non-referral, other reasons specific to developing country contexts include insufficient resources such as lack of diagnostic equipment in most state hospitals, as well as insufficient numbers of audiologists in the country. Swanepoel (2006) described the major challenges to the provision of adequate audiological services in South Africa as being attributed to an insufficient number of audiologists which is unequally distributed between the private and public sector. Lack of referral may also indicate the differential priorities placed on presenting symptoms in any admitted child; that is, hearing loss may not be viewed as a priority during the early medical management. Non-referral of paediatric cases following meningitis has serious consequences for early detection of hearing loss and subsequent rehabilitation which may minimize the almost inevitable speech and language difficulties, academic difficulties, as well as social and emotional maladjustment that come with a hearing impairment.

When it came to the timing of referrals for audiological services, a second aim of the current study, findings indicate that almost all the cases that were referred (80%) were done so as in-patients before discharge from the hospital; with the remaining cases being referred within three months after discharge. These findings are consistent with previous reports which indicate that a large number of cases are referred before discharge. For example, in a retrospective audit of case notes of children notified to the Public Health Department of Wales, 50% of children were assessed before discharge with 30 % being assessed within the first 8 weeks following infection, while the remaining 19% had no record of hearing assessment (Wilson et al., 2003). Because in the current study no cases were referred after three months, it may be inferred that if patients are not referred as in-patients or within three months following discharge from hospital, they may never be referred for audiological assessment. This has implications for paediatricians managing these cases in that in-patient referral for hearing tests may need to be prioritized with a follow up appointment arranged as part of the medical follow up clinic. The in-patient referral is also important because researchers such as Woolley et al. (1999) assert that in a majority of cases, if hearing loss is present at discharge from hospital, recovery of hearing does not occur, hence early identification and management of this hearing loss becomes crucial. The follow up audiological assessment would then ensure that middle ear deafness that Dodds, Tyszkiewicz and Ramsden (1997) report only resolves 2–4 weeks after discharge, can also be taken into account and hearing function monitored. This referral pathway, if well-defined, can improve assessment/referral rates post meningitis as advocated by Wilson, Roberts and Stephens (2003).

Referrals for audiological services by paediatricians need to be met with appropriate and accurate audiology measures that allow for reliable and valid results to be obtained. Findings from the current study were disturbing in as far as audiological tests conducted were concerned. It is of great concern that of all the cases referred for hearing tests, no diagnostic testing (in the form of pure tone audiometry, speech audiometry, auditory brainstem response, and auditory steady state responses) was conducted. Only nine cases had OAEs performed, with the rest of the sample only having otoscopy and tympanometry. These findings could have been influenced by non-standardized record keeping protocols, an acknowledged limitation of the current study design. For example in some hospitals, audiology records are maintained separate from the hospital file, and going through these files as well could have yielded different results. This highlights the need for standardization of record keeping processes which may have significant medico-legal implications as well as implications for assurance of continuity of care (Hamilton et al., 2003). Woolley et al. (1999) advocate that OAEs combined with tympanometry followed by an ABR, if indicated, is the best test battery for the paediatric meningitis population. Furthermore, these authors emphasize the importance of conducting behavioural audiometry as soon as possible; in this way the degree and configuration of the hearing loss may be established (Woolley et al., 1999; Faraji et al., 2004). The fact that nine of the cases in the current study had OAEs performed is positive since studies on the efficacy of OAEs as a screening tool for children recovering from meningitis screening have found that OAEs are both feasible and effective (Faraji et al., 2004). These authors reported that the technique of OAEs was found to be well tolerated by patients and was considerably easier to perform than ABRs. Furthermore, OAEs were found to be highly sensitive and relatively specific and therefore in-patient OAE screening should assure early diagnosis of hearing loss and prompt auditory rehabilitation (Faraji et al., 2004). Within a developing country with limited resources, it would seem that investing in OAE equipment for all paediatric wards would be a sound investment.

As far as determination of a correlation between signs and symptoms of meningitis and audiology referrals was concerned, the current study found fever to be the only significant predictor variable. This infers that if one were to model predictors that could determine whether a child will be referred for an audiological assessment in the current study, fever was the only variable indicator. Therefore, fever must be acknowledged as a crucial factor to be included during the development of audiology referral protocols post meningitis. This finding is particularly important where targeted hearing screening may be the only option available to audiologists due to limited resources. Fever has been previously reported as a predictor variable for hearing loss in children with bacterial meningitis (Kutz et al., 2006); hence it is possible that in the current study these were the patients that were referred because they presented with an obvious hearing impairment. Other predictor factors such as elevated cerebrospinal fluid (CSF) protein and decreased cerebrospinal fluid glucose (Kutz et al., 2006) were not explored in the current study and so it is not possible to say if CSF results would have been found to be a predictive factor since they did not form part of the logistic regression analysis, an acknowledged study design limitation.

It is important to note additional findings from the current study which reveal that significant referrals were made to the allied medical disciplines including physiotherapy, occupational therapy, speech-language therapy, as well as dieticians. These professionals can be targeted by audiologists as part of the referral pathway for audiological services for children post meningitis. Audiology referral rates may be increased by providing these professionals with information regarding risk factors for hearing loss, and presentation of hearing impairment. In this way, children who were not referred for audiology services as in-patients may be referred by allied medical disciplines on follow up visits. This, however, does not take the responsibility away from the doctors who should be the primary referring agents to the audiologists. The collaboration among professionals and families in the effective delivery of services to infants and children with hearing loss is imperative in ensuring that appropriate services are provided.

Additional findings also indicated that the average duration of hospital stay in the current study was 10.3 days ±4.4 days. The shortest duration of stay was 5 days and the longest 21 days. In a study on hospitalization during bacterial meningitis in Canada the overall median length of stay (LOS) was 11 to 12 days and remained stable over the study period from the year 1994 to 2001 (Canada Communicable Disease Report, 2005). It must be noted though that in this Canadian study, the duration of hospital stay varied by organism from 8 to 21 days. Although, the duration of hospital stay is not directly related to hearing loss or significantly associated with future hearing loss, as revealed in a study by Wolley et al. (1999); this information was thought to be of great relevance to the South African audiologist as the duration of hospital stay may present a challenge to the audiologist in terms of the development of audiology referral protocols under resource constraints. Audiologists need to be aware of the lack of consistency regarding the duration of hospital stay as this indicates the necessity for highly individualized assessment and treatment plans; that ensure that children are not discharged before a referral for audiology is made.

## Conclusions

This study investigated the audiological referral protocols of populations post meningitis by means of a retrospective record review. The results of this study indicated that a significant number (40%) of cases were not referred for audiological assessment. The majority of the cases in the current study were referred as in-patients with very minimal referral occurring after discharge from the hospital. Those cases referred did not seem to have undergone comprehensive diagnostic audiological assessment with all cases only having otoscopy and tympanometry conducted, and only 9 having OAEs performed. The only variable that was a significant indicator for audiological referral in the current study was fever, although it is acknowledged that the list of variables included in the logistic regression analysis in the current study was not exhaustive.

These findings highlight the need for establishment of referral protocols for this population with specific referral pathways drawn up, specific times of referrals, as well as specific audiological measures that need to be employed to ensure that the adverse consequences of hearing impairment following meningitis are minimized. These findings also indicate the need for the provision of training for allied medical disciplines as well as medical disciplines regarding risk factors for hearing loss which may improve referral rates for early detection and intervention of hearing impairment. The current study seems to indicate that improved hearing assessments of children following meningitis may be achieved if patients are referred while they are still in-patients with OAEs being an important tool in the battery of tests that need to be available in all paediatric wards.

The current study did however have limitations including that of the fact that a small sample size from only two academic hospitals was recruited; hence generalization of the findings to other sites may be difficult. However, referrals may even be poorer in the primary and secondary level health care systems where resources are possibly more restricted. Furthermore, the possibility of findings being influenced by the methods of record keeping used at the two research sites should also be acknowledged. Lastly, because this study was based on record reviews, factors influencing referrals could not be explored; and these are important to document so that remediation strategies can be put in place; hence implications for future research where these actors can be taken into consideration on larger sample sizes.
